# Orthographic Networks in the Developing Mental Lexicon. Insights From Graph Theory and Implications for the Study of Language Processing

**DOI:** 10.3389/fpsyg.2018.02252

**Published:** 2018-11-20

**Authors:** Jutta Trautwein, Sascha Schroeder

**Affiliations:** Max Planck Research Group (MPRG) Reading Education and Development, Max Planck Institute for Human Development, Berlin, Germany

**Keywords:** mental lexicon, networks, orthographic neighborhood, graph theory, reading development

## Abstract

In this study, we examine the development of orthographic networks in the mental lexicon using graph theory. According to this view, words are represented by nodes in a network and connected as a function of their orthographic similarity. With a sampling approach based on a language corpus for German school children, we were able to simulate lexical development for children from Grade 1–8. By sampling different lexicon sizes from the corpus, we were able to analyze the content of the orthographic lexicon at different time points and examined network characteristics using graph theory. Results show that, similar to semantic and phonological networks, orthographic networks possess small-word characteristics defined by short average path lengths between nodes and strong local clustering. Moreover, the interconnectivity of the network decreases with growth. Implications for the study of the effect of network measures on language processing are discussed.

## Introduction

The study of the structure of the mental lexicon and its effect on lexical access has been of interest for several authors in the past. However, although it is unquestionable that orthographic neighborhoods affect word processing during reading development, the development of orthographic similarities in the mental lexicon has rarely been analyzed. The purpose of this study is to examine the development of orthographic similarities in the mental lexicon during reading acquisition by applying graph theory to simulated data of lexical development. We first highlight the importance of orthographic knowledge in reading development and then define neighborhoods in different language domains as well as their effect on language processing, also regarding developmental changes in effects. Subsequently we report studies on the development of neighborhoods in the mental lexicon and point out that there is a lack of studies on orthographic development. Afterward we present graph theory and its advantages to examine the neighborhood structure of the mental lexicon. Before presenting our methods and results on the development of orthographic networks, we describe the necessity of simulated data for the age group of interest. In the Discussion section, we relate our findings to current theories of orthographic development and effects of neighborhoods on visual word recognition. In addition, we discuss implications for future studies of language processing during lexical development.

### Orthographic Knowledge in Reading Development

Sophisticated orthographic knowledge is crucial for reading competence. When decoding print, we compare the read word form with words already stored in our orthographic lexicon which allows us to access semantic information as well as phonological information for reading out loud. This process is implemented in models of the reading process and is necessary for an efficient reading competence (e.g., the DRC, Coltheart et al., 2001). During reading development, a learner has to establish and improve his or her orthographic lexicon as well as the process of word retrieval from it. In models of reading development it is assumed that children shift from letter-by-letter reading to a more word-based process of lexical access, probably because more and more words are stored as a whole in their orthographic lexicons (Acha and Perea, 2008). Castles et al. (2007) suggest that the recognition process shifts from a more broadly to a more finely tuned mechanism and support their theory with form-priming experiments. They showed form-priming effects for developing readers but no effects for proficient readers. Their explanation involves the composition of the orthographic lexicon: Beginning readers only know a few words that are similar to the form prime. That is, the form prime eases activation of the target word. Proficient readers, however, know a lot of words similar to the form prime and so activation cannot concentrate on the target word alone. That is, in this framework, orthographic similarities within the orthographic lexicon play an important role in the reading process.

### Neighborhoods in the Mental Lexicon: Structure and Effects

The mental lexicon comprises information on phonological, orthographic and semantic features of words. It is assumed that entries are interconnected due to shared features in these domains (Perfetti and Hart, 2002). That is, the mental lexicon can be conceptualized as a large network with nodes and connections. Directly connected words are usually referred to as *neighbors*. Semantic neighbors are words with similar semantic characteristics (e.g., *salt* – *pepper;* Aitchison, 2012). Phonological neighbors are defined as words that can be created by exchanging, deleting or adding one phoneme from another word (e.g., *cat /kæt/– hat /hæt/*; Yates, 2005). Similarly, orthographic neighbors are defined as words that can be created by exchanging, deleting, or adding a single letter from another word. Since the mental lexicon grows in size during language development (Segbers and Schroeder, 2017), the neighborhoods within the mental lexicon might also change.

The study of neighborhoods in the mental lexicon is particularly interesting because neighbors have shown to influence language processing. For example, semantic neighbors often ease processing of target words in semantic priming experiments (e.g., Sánchez-Casas et al., 2006; Holderbaum and Fumagalli de Salles, 2011), although some studies also show an inhibitory effect depending on nature of semantic relation between prime and target (Abad et al., 2003; for a review see Neely, 2012). Furthermore, it has been shown that words with a lot of semantic neighbors can be retrieved faster (e.g., Buchanan et al., 2001), that is the effect of semantic neighbors is facilitative. For phonological neighbors, a study by Yates (2005) has led to similar results with a facilitative effect of phonological neighbors on visual word recognition. Mulatti et al. (2006) also found this effect for reading aloud. In this framework, the activation of neighbors boosts the activation of the target word. For orthographic neighborhoods, the results on the effect are controversial as summarized in the review by Andrews (1997). Although many studies also found facilitative effects, Andrews (1997) points out that the frequency of the neighbors also have an important influence. That is, the presence of high frequency neighbors inhibits the access of low frequency target words (see also Grainger, 1990; Sears et al., 1995; Grainger and Jacobs, 1996; Pollatsek et al., 1999; Grainger et al., 2005). In this framework, the activation of high frequency neighbors impedes the activation of the target word since they compete with each other.

### Developmental Changes in Neighborhood Effects and Lexical Structure

Only a few studies addressed developmental changes in the effect of neighborhoods on word recognition. Holderbaum and Fumagalli de Salles (2011) found higher semantic priming effects for children in visual word recognition than for adults. This indicates that they rely more on semantic information than skilled readers, probably because their orthographic lexicon is still developing. For phonological neighborhoods, Metsala (1997) found developmental differences in the processing especially for words from sparse neighborhoods and low frequency words. He ascribes these findings to developmental changes in the mental lexicon which is refined during language acquisition. Castles et al. (2007) examined masked form-priming using orthographic primes that either differed in one letter from the target word (*rlay – play*) or where two letters were transposed (*lpay – play*). They found priming effects only for beginning readers which dissolved during development and also attributed these findings to a shift from a more broadly to a refined processing mechanism. Thus, the composition of the orthographic lexicon in terms of neighborhoods is directly linked to the development of reading competence. That is, although the number of studies analyzing developmental changes in neighborhood effects is limited, they all ascribe their results on developmental patterns to changes in the mental lexicon and its access during language and reading acquisition.

To some extent, developmental trajectories in lexical development have been assessed. For example, for semantic neighborhoods in the mental lexicon, Steyvers and Tenenbaum (2005) could show that new words enter the lexicon when they already have a lot of neighbors in the vocabulary. However, Hills et al. (2009) also tested further developmental mechanisms that might drive semantic neighborhood development. They conclude that words with many semantic neighbors in the learning environment are more noticeable and represent key words in the network which makes them important. Similar results have also been found in several further studies investigating semantic networks (Hills et al., 2010; Bilson et al., 2015). For phonological neighbors, a similar pattern has been reported. In particular, Storkel (2004) showed that age of acquisition and phonological density influence phonological neighborhood growth and that words from dense phonological neighborhoods are learned earlier. Vitevitch and Storkel (2012) found the same pattern using computational models of network learning. Further evidence comes from Stamer and Vitevitch (2012) who showed that words from dense phonological neighborhoods are acquired earlier in second language learning. The only evidence for developmental changes in orthographic neighborhood size has been provided by Castles et al. (1999) who, however, used a completely different approach. They selected words with a high and a low orthographic neighborhood size and presented the target words as well as the neighbors on a list together with nonwords to children and adults. The participants were asked to identify all words they know out of the list. The authors considered identified words as existing neighbors in the participant’s lexicon and called this measure their “effective neighborhood size.” They then compared knowledge of neighbors of children and adults for words with dense and sparse neighborhoods. Importantly, children knew fewer neighbors than adults for words with dense and sparse neighborhoods. This is in line with the notion that children’s effective neighborhood size is small for all words. Further analyses on orthographic network development are still missing. However, to understand and predict effects and developmental changes of neighborhoods during language acquisition, those analyses are necessary.

To sum up, the study of neighborhood effects in lexical access has been of interest in several different approaches, also regarding developmental changes. They are often ascribed to developmental changes in neighborhoods in the mental lexicon and are connected to changes in reading development. However, these developmental changes have not been determined for orthographic development yet. That is, the properties of orthographic networks are still unclear. However, since orthographic neighbors influence orthographic processing (see Andrews, 1997), the examination of the neighborhood structure and its influence on reading and writing is highly important. The aim of this study is thus to shed light on the courses of lexical development regarding orthographic neighborhoods. Results could be used to predict and explain effects of neighborhoods in reading development and processes in reading acquisition.

### Analyzing Networks Through Graph Theory

One approach to investigate connections in the mental lexicon and their development is graph theory. It has been used in some studies in order to analyze semantic (Steyvers and Tenenbaum, 2005; Zortea et al., 2014) and phonological networks (Vitevitch, 2008; Chan and Vitevitch, 2010) and is also applicable to other fields of network research such as brain interconnectivity in neuroimaging (e.g., Rubinov and Sporns, 2010; Van Wijk et al., 2010). Besides, network models can be used to identify conditional (in-)dependencies between variables or competencies, e.g., with regard to reading ability (Colé et al., 2018).

According to the graph approach concerning the mental lexicon, words are represented as nodes and connections (= neighborhoods) as paths between nodes. Several measures can be used to describe the network. The number of nodes *n* represents the number of words in the mental lexicon. The number of links of a node *k*_i_ is equal to the number of direct neighbors and is also referred to as the degree. It can be averaged across the whole network with <*k*>. The distribution of the degree *P*(*k*) represents the probability that a randomly chosen node has the degree *k* and is thus another measure of connectivity of the network. The average path length *L* and the maximum path length between two nodes (also referred to as the diameter) *D* represents the number of steps needed to get from one node to the other. The clustering coefficient *C* measures the probability that two neighbors of a node are neighbors themselves and is thus a measure of graph connectivity (Steyvers and Tenenbaum, 2005).

For all these measures, words that are not connected to the network are excluded. However, these so-called “lexical hermits” also provide information on how well the network is interconnected and should be considered as well. Thus, all measures yield information on the interconnectivity of the network which might influence language processing. They thus can be regarded as an extension of the traditional neighborhood measure.

For phonological networks, Chan and Vitevitch (2009, 2010) already demonstrated the use of network measures to analyze neighborhood effects in language processing above the traditional measure of neighborhood size. In this study, we will thus determine network measures for orthographic networks during lexical development. Furthermore, network measures allow the comparison of networks in different (language) domains. Several studies have shown that many networks possess small world characteristics (Watts and Strogatz, 1998). That is, they exhibit a high interconnectivity between nodes as indicated by a short average path length and a high clustering coefficient. Furthermore, such networks have a scale-free structure with a power-law degree distribution. This means that few nodes have many connections while many nodes only have few connections. This structure appears to be ideal for language processing since it allows a high local interconnectivity (= clusters) as well as easy global access through “bridges” that connect clusters (Beckage et al., 2011). Steyvers and Tenenbaum (2005) as well as Hills et al. (2009) demonstrated that this also holds for semantic networks in natural language. In addition, Vitevitch (2008) showed that this finding generalizes to phonological networks in English as well as in other languages (Arbesman et al., 2010). For orthographic networks, no comparable studies have been conducted yet. In particular, it is unclear at present, whether orthographic networks possess small-world characteristics and a scale-free structure similar to other language domains. Another aim of this study is thus to examine, whether orthographic networks are structured similar to other language domains with small world characteristics.

### Challenges of Lexical Measurement

To examine changes in orthographic neighborhoods, it appears to be reasonable to analyze mental lexicons of children during orthographic development. During this phase, children add a lot of new entries to their vocabulary (Segbers and Schroeder, 2017), thus great developmental differences can be expected. In addition, several authors have assumed a lexical restructuring from a broader to a more fine grained access process in this phase (e.g., Castles et al., 2007) which could also be due to developmental changes in lexical content. We thus decided to analyze orthographic lexicons for children from Grade 1 to 8.

However, the measurement of vocabulary and thus orthographic networks is challenging. While the number of known words in young children is limited and thus relatively easy to estimate, the orthographic lexicon grows rapidly after children enter school (Anglin et al., 1993; Segbers and Schroeder, 2017). As a consequence, it is impossible to analyze the complete vocabulary by testing every word a child might possibly know. However, one way to approximate the size and content of children’s orthographic lexicon is the dictionary method (e.g., Nation, 1993). In this method, words sampled from a dictionary are tested and then the results are projected onto the whole lexicon. Using a variant of this method, we (Segbers and Schroeder, 2017) have been able to estimate the average vocabulary size in grades 1–8 in German. In that study, word frequency was used as a proxy variable to simulate language learning, although other factors might also influence language development (see Discussion section). In the present study, we wanted to add to these findings and further analyzed the structure of the orthographic networks and the development of their characteristics. Since lexicon size increases dramatically between grades 1 and 8 (Segbers and Schroeder, 2017) we assumed a change in lexical structure supporting an enhancement of language processing for an improving, more efficient reading process. Since network characteristics have been shown to influence language processing, the findings were aimed to lead to implications for further research on the effect of network measures on lexical access.

For this purpose, we used the average vocabulary sizes per grade to simulate data on the content of vocabularies for 50 virtual participants. We did this by using the childLex copurs, a written language corpus which represents the reading environment of German children aged from 6 to 12 (Schroeder et al., 2015b). By sampling words from the corpus we conducted the content of lexical development for 50 virtual participants. As in the former study (Segbers and Schroeder, 2017) we used word frequency as a proxy variable which drives language learning. This enabled us to analyze the simulated data in terms of network measures and their development with growing lexicon size.

## Materials and Methods

### Sampling Procedure

We simulated 50 prototypical language learners in German who we subsequently refer to as virtual participants. The question whether effects are significant is less important in simulation studies because sample size can be arbitrarily increased. Instead, it is more useful to focus on overall developmental differences and the shape of the effects. As a consequence, the present sample size was chosen so that medium to large effects (*r*∼0.3–0.4) could be detected with a power of 0.80 using a significance level of α = 0.05.

Sampling was based on the childLex corpus (version 0.16, Schroeder et al., 2015b) which is a corpus consisting of 500 German children’s books for a reading age from 6 to 12 years. It is thus representative for children’s reading environment when they start to read. The complete corpus was treated as the fully developed adult network. The childLex corpus comprises ca. 10 million tokens which are distributed over approximately 180,000 types (distinct word forms including inflection etc.) and 120,000 lemmas (syntactic base forms; see Schroeder et al., 2015b, for details). As linguistic networks are typically analyzed on the type level, we used types (distinct orthographic sequences) in the following analysis (see Table 1). However, analyses on the lemma level lead to a very similar pattern of results concerning developmental changes. They are depicted in Supplementary Table 1 of the online appendix. The sampling procedure was sensitive to type frequency, i.e., types that occur more often in the corpus were more likely to be drawn.

**Table 1:** Lexicon sizes and network measures in different age groups.

	*M* lexicon size^∗^		Network measures *M (SD)*					Lexical hermits *M* (*SD*)	
			
Grade	Lemmas	Types	*n*	<*k*>	*L*	*D*	*C*	*n*	Proportion
1	5,925	31,570	16,027 (84)	6.78 (0.06)	9.84 (0.29)	46.04 (5.51)	0.49 (0.02)	15,543 (84)	49%
2	6,097	32,606	16,580 (86)	6.79 (0.06)	9.85 (0.30)	45.44 (5.37)	0.49 (0.02)	16,026 86)	49%
3	11,182	46,757	24,155 (87)	6.90 (0.05)	9.99 (0.28)	49.22 (6.45)	0.47 (0.01)	22,602 (87)	48%
4	14,819	58,238	30,368 (94)	6.93 (0.04)	10.25 (0.25)	51.64 (6.24)	0.45 (0.01)	27,870 (94)	48%
5	18,812	71,344	37,479 (115)	6.95 (0.04)	10.52 (0.19)	51.30 (4.92)	0.43 (0.01)	33,865 (115)	48%
6	25,694	93,293	49,465 (118)	6.96 (0.02)	10.56 (0.13)	47.48 (3.35)	0.41 (0.00)	43,828 (118)	47%
8	38,029	130,675	70,123 (109)	6.98 (0.02)	10.51 (0.08)	45.18 (3.86)	0.38 (0.00)	60,552 (109)	46%


*^∗^*Lexicon sizes were determined via a sampling procedure established by Segbers and Schroeder (2017). In this framework, we developed a vocabulary test (Trautwein and Schroeder, 2018). We then determined the relation between vocabulary test results and total vocabulary size by drawing a virtual sample of different lexicon sizes from the childLex corpus and checking whether the items of the vocabulary test were included or not. We then adapted this relation to a sample of real children and determined their total vocabulary sizes for different grades.**

We assumed that there were no differences in the overall size of the mental lexicon between children. That is, the size of the networks of all 50 virtual participants in each grade were identical and corresponded to the average grade-specific lexicon size reported by Segbers and Schroeder (2017) which are provided in Table 1. The sampling procedure worked as follows: The estimated average lexicon size in grade 1 is 31,570 types. In a first step, we sampled 31,570 types from the childLex corpus for each of the 50 virtual participants. This set represented the initial state of their orthographic network and was different for each virtual participant. As the sampling procedure was sensitive to frequency, high-frequency types (function words, etc.) were likely to be included in all virtual lexicons. However, because the virtual lexicons were sampled independently, they also differed from each other – particularly in the low-frequency range.

After this initial sampling step, all remaining types in the childLex corpus (i.e., 180,000–31,750) were used as the basis for the second step of the sampling procedure which represents the growth of the orthographic lexicon between grade 1 and 2. This set represents the learning environment, i.e., it comprises all words in children’s print environment that are still left to be learned. As the initial lexicons differed between virtual participants, their learning environments were also different. Again, the sampling procedure was sensitive to word frequency, i.e., words which were more frequent in the learning environment were more likely to be drawn.

**FIGURE 1:**
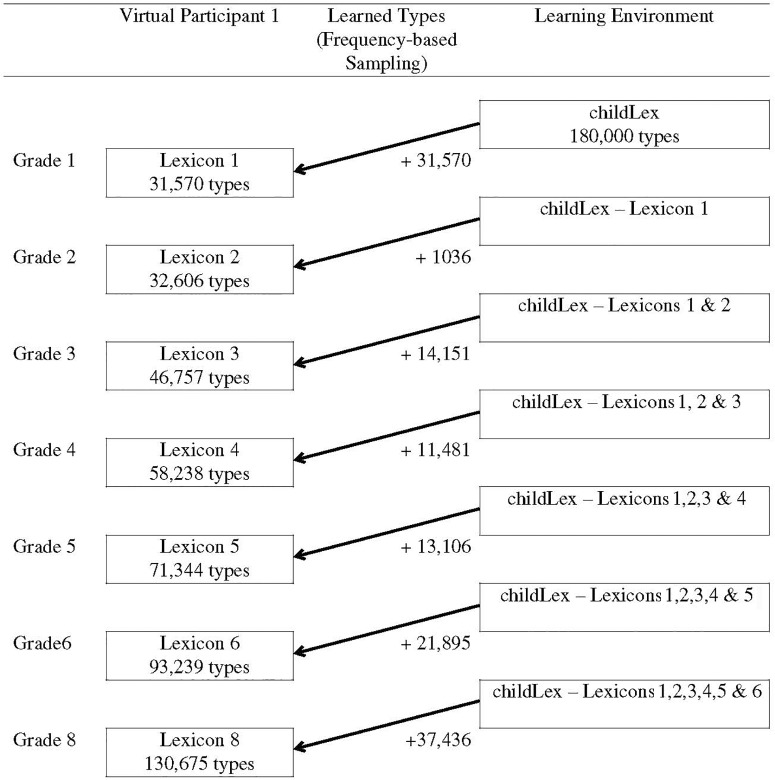
Schematic Illustration of the sampling procedure for one virtual participant.

In a next step, 1,036 new types were added to each of the 50 virtual lexicons. This number is the difference between the average size of the lexicon in grade 1 (31,750 types) and the average size of the lexicon in grade 2 (32,606; see Table 1). The number of newly learned words was the same for all virtual participants and we thus assumed that there were no differences in the rate of lexicon growth. The new types were sampled from the individual learning environment of each virtual participant and the sampling procedure was again sensitive to frequency. Figure 1 schematically illustrates the sampling procedure for one virtual participant. The right column shows the learning environment at each time point: A the beginning, the total childLex corpus was used for sampling. For the following steps, the already learned words were excluded from the learning environment since they don’t need to be learned anymore. The middle column shows the number of learned words for each time point. The left column illustrates the particular lexicon size on the type level for each grade.

This same procedure was repeated for the following grades (3, 4, 5, 6, and 8) until the lexicon had the size of the average language learner in grade 8. This resulted in seven orthographic lexicons for each of the 50 virtual participants (grades 1–6 and 8), that is, 350 lexicons in total. We compared the mean frequencies of the newly learned words in each age group with the mean frequencies of words per grade as determined by Segbers and Schroeder (2017). Both frequency trajectories showed a decreasing pattern, i.e., high-frequency words were acquired first and low-frequency words later. This pattern is also observed in the childLex corpus (see Schroeder et al., 2015b) indicating that the sampling procedure of the present study reflects the actual learning process of children in language acquisition.

### Analyses

We computed important network characteristics for each grade-specific lexicon of each virtual participant. These analyses were performed with the igraph (Csardi and Nepusz, 2006) and the vwr package (Keuleers, 2013) in R. For each virtual participant in each grade, an unweighted orthographic network was created in which types served as nodes and were connected with each other via paths if they were orthographic neighbors.

Traditionally, orthographic neighbors have been defined by the substitution of a single letter (Coltheart et al., 1977). More recent approaches, however, assume that words created via deletions or insertions are also orthographically related (Yarkoni et al., 2008). Our definition of orthographic neighbors is thus based on the orthographic Levenshtein distance, a measure to quantify the (dis)similarity between two letter strings which takes substitutions, deletions, and insertions into account (Levenshtein, 1966). Orthographic neighbors were defined as words with a Levenshtein distance of 1, i.e., words that can be created by substituting, deleting, or inserting a single letter in a source word (e.g., for the word “hat”, not only words such as “hot” are neighbors but also words such as “hate”).

Following Vitevitch (2008) “lexical hermits” (i.e., nodes that do not connect to any other node in the lexicon) were excluded for the construction of the networks since some of the graph measures can only be applied to fully connected networks (also see Discussion for the role of lexical hermits). The number and proportion of lexical hermits were determined. For each network, we calculated the graph measures (for the explanation see also Introduction) *n* (number of nodes in the network), <*k*> (mean degree, that is the mean number of neighbors per word), *P*(*k*) (distribution of degree, that is the distribution of degrees for the whole network), *L* (average path length, that is the average number of paths to get from one node to another), *D* (maximum path length, that is the maximum number of paths to get from one node to another) and *C* (clustering coefficient, that is the probability of neighbors of a word to also be neighbors). *C* is calculated over all nodes *i* using the formula

(1) $$C_{i}=T_{i}/(K_{i}/2)=2T_{i}/k_{i}(k_{i}-1)$$

*T*_i_ can be referred to as the number of links between the neighbors, *k* of the node *i* and *k*_i_ (*k*_i_ -1)/2 stands for the number of connections that would be assumed if all neighbors of a node were also neighbors (Steyvers and Tenenbaum, 2005). Each network measure served as the outcome variable in a repeated measurement ANOVAs using grade (1, 2, 3, 4, 5, 6, 8) as a factor varying within virtual participants (which also stands for varying network size). Significant overall effects were complemented by computing t tests (using Tuckey’s correction for multiple comparisons) between consecutive grades. In addition, the shape of the overall effect of grade on each network measure was analyzed by fitting different functions to the data.

## Results

**FIGURE 2:**
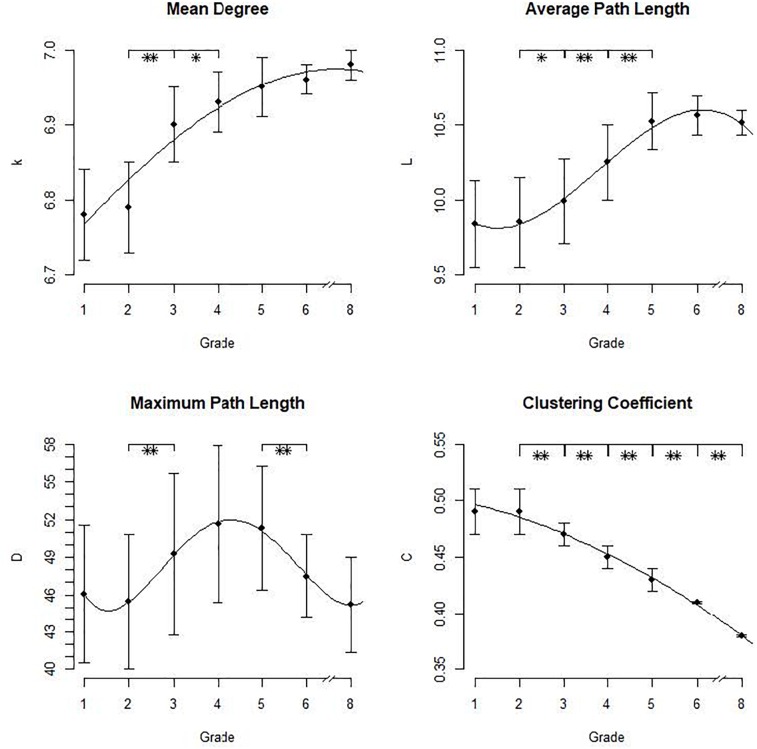
Means and standard deviations for the network measures in the different grades with overall effects depicted as lines. ^∗^*p* < 0.05, ^∗∗^*p* <0.01.

In our analyses we were interested in the properties of orthographic lexical networks and how they develop over time in our virtual simulation of children’s reading acquisition. Important descriptive statistics for the network measures in each grade are provided in Table 1. The developmental patterns for these measures are displayed in Figure 2. Lines represent the shape of the effect of grade on each measure.

The mean degree of the network’s nodes <*k>* increased with age, *F*(6,343) = 164.8, *p* < 0.001. Thus, the number of neighbors per node increases overall as expected. The results showed a mean number of orthographic neighbors between 6 and 7 across all age groups. *Post hoc* analyses showed a significant difference only between second and third grade, *p* < 0.001, and third and fourth grade, *p* < 0.05. The shape of the effect could be described with a quadratic function indicated by a significant linear trend = -0.005, *t* = -7.66, as well as a significant quadratic trend = -0.079, *t* = 13.28 (intercept = 6.69, *t* = 637.77). With an *R*^2^= 0.70, the fit of the function was sufficient, that is it adequately represents the data. The curve thus shows a deaccelerating trend with a strong increase of neighbors in the beginning which levels out later in development after fifth grade. That is, overall, the number of neighbors per node increases with network development, mostly between second and fourth grade where a lot of new neighbors are added to the existing network. From grade 5 onwards, the development is slower: although the number of neighbors still increases, the growth grade becomes smaller.

The average path length *L* ranged between 9.84 and 10.56, that is between 9 and 11 paths were necessary to get from one node to another. The effect of grade was significant, *F*(6,343) = 96.92, *p* < 0.001. *Post hoc* analyses revealed a significant increase between second and third grade, *p* < 0.05, between third and fourth grade, *p* < 0.001, and between fourth and fifth grade, *p* < 0.001. The effect of grade on the average path length could be described by a cubic function indicated by a significant linear trend = -0.016, *t* = -7.08, a significant quadratic trend = 0.18, *t* = 6.69 and a cubic trend = -0.44, *t* = -4.54 (intercept = 10.11, *t* = 106.17). The fit of the function was sufficient, *R*^2^ = 0.63, indicating an adequate representation of the data. That is, in the beginning the development of the average path length is weak, from second to fifth grade it increases rapidly and afterward the curve levels out. Again, we observed a deaccelerating trend with larger differences in the early grades and smaller increases in later development. Overall, the growth of the average path length indicates that the interconnectivity of the network decreases with growth since more paths are necessary to get from one node to another. The loss of interconnectivity is thus strongest between second and fifth grade. Afterward, the average path length still increases but with a decreased growth rate. Overall, that is, although the number of neighbors increases, the interconnectivity of the orthographic network decreases.

The maximum path length *D*, that is the maximum number of paths between two nodes, ranged between 45 and 52. From first to second grade, the diameter decreased, then increased until fourth grade and decreased again up to eighth grade. The overall effect of grade was significant, *F*(6,343) = 13.58, *p* < 0.001. *Post hoc* analyses, however, showed a significant increase only between second and third grade, *p* < 0.01, and a decrease between fifth and sixth grade, *p* < 0.01. The shape of the effect could be displayed as a quartic polynomial (linear trend = 0.13, *t* = 3.68, quadratic trend = -2.17, *t* = -3.92, cubic trend = 12.03, *t* = 3.95, quartic trend = -23.41, *t* = -3.57, intercept = 59.47, *t* = 13.35) but the fit of the function was very low (*R*^2^ = 0.19) in comparison to the fits of the other functions (all *R*^2^> 0.6). That is, the function did not sufficiently represent the data. Thus, concerning the diameter, we could not obtain a clear pattern of network development since a rising diameter means a loss of interconnectivity but a decreasing diameter means a higher degree of interconnectivity.

**FIGURE 3:**
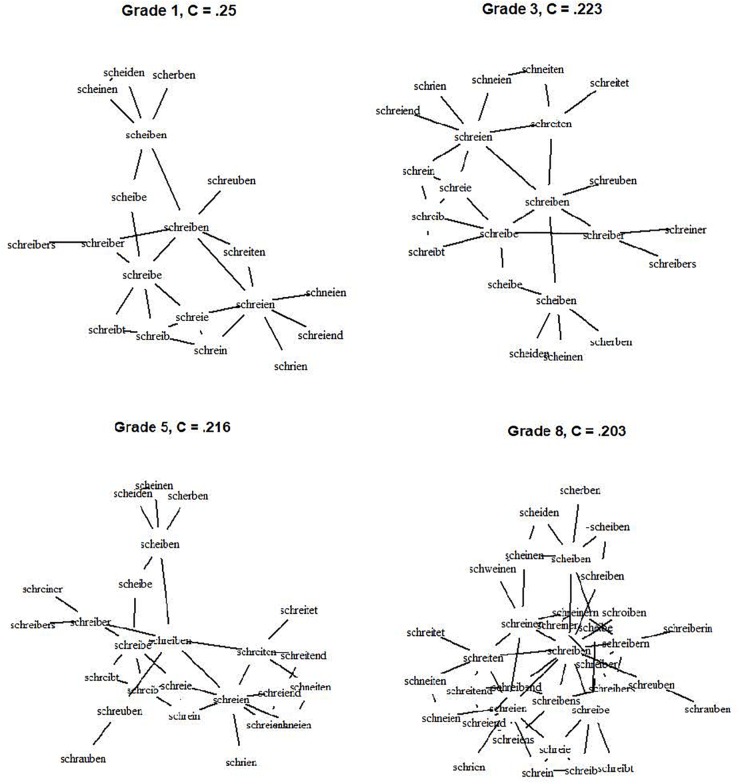
The network and according clustering coefficient for the word “schreiben” – “to write.” Note that for reasons of comprehensibility only neighbors of a maximal Levenshtein distance of 2 are depicted.

The clustering coefficient *C* ranged between 0.38 and 0.49 which indicates a high probability of the neighbors of a word to also be neighbors of each other. The effect of grade was significant, *F*(6,343) = 631.8, *p* < 0.001. While the difference between first and second grade was not significant, *p* > 0.90, *C* decreased from second grade onwards, all *p* < 0.01. The shape of the effect could easily be described as a quadratic function evident via a significantlinear trend = -0.002, *t* = -8.43 and a significant quadratic trend = -0.007, *t* = -4.205 (intercept = 0.51, *t* = 185.04). The fit statistic of the function was very high, *R*^2^ = 0.99, indicating that the quadratic function adequately describes the data. The curve shows a small decrease in the beginning with no significant difference between first and second grade and a steady strong decline of the clustering coefficient onwards. We thus observe an accelerating trend with small differences in the beginning of lexical development and larger differences later in development. To illustrate this, an excerpt of the growing network is depicted in Figure 3. It shows the network of the word “schreiben” (“to write”) with neighbors of a maximum Levenshtein Distance of 2. As evident, more neighbors are added but the clustering coefficient decreases. Overall, the decrease of the clustering coefficient indicates that with network growth, the probability of neighbors to also be neighbors decreases. Thus, with growth, the interconnectivity of the network declines, especially from grade 2 onwards.

The number and proportion of lexical hermits, that is words that do not relate to the lexical network, are also displayed in Table 1. While the total number of hermits increases with lexical growth, their proportion in relation to the total lexicon stays constantly high at almost 50% for all age groups. That is, a high number of words is not connected to the mental lexicon at all.

**FIGURE 4:**
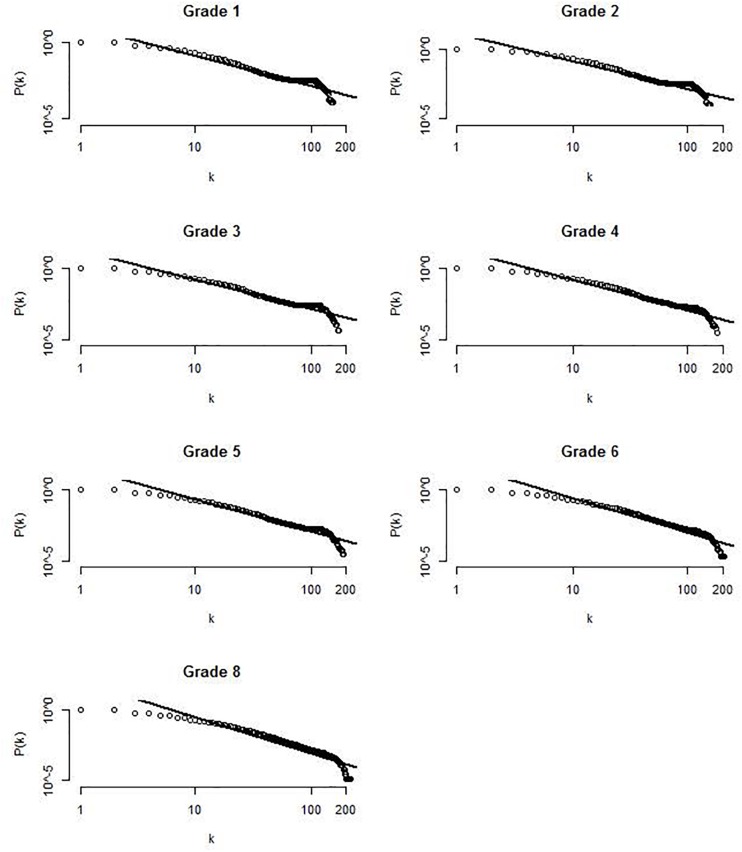
Log-log plot of the degree distribution for one exemplary virtual participant at each time points. Lines represent the fit of a linear function to the data.

Another important question is whether the number of links between the nodes follows a power-law distribution similar to networks in other language domains. This is typically demonstrated by plotting *k* against its relative frequency *P*(*k*) on a log-log scale. If this relationship is linear, the network has a structure that follows a power-law distribution. The degree distribution for one exemplary virtual participant for each grade is displayed in Figure 4. As can be seen, the relationship between log *k* and log *P(k)* appears to be linear. A linear function provided a good fit of *R*^2^ > 0.85 in for all virtual participants at each time point.

That is, the degree distribution follows a constantly descending power-law function and the number of nodes with a lot of connections decreases.

To sum up, the network measures show highly interconnected orthographic networks in orthographic development. However, although the number of neighbors per node slightly increased with development, the interconnectivity of the network decreased with network growth. Apart from the diameter, this pattern could be obtained in all network measures. The proportion of hermits stays constant throughout development. Furthermore, there are a lot of nodes with a few connections and only a few nodes with a lot of connections as shown by the descending function of the degree distribution.

## Discussion

In this study, we analyzed orthographic networks and their development from grade 1 to grade 8 in German. With a frequency-sensitive sampling approach, we simulated orthographic lexical development for 50 virtual participants from grade 1 to 8 and examined their networks’ characteristics and their development by using graph theory. Findings indicate that the number of orthographic neighbors per word increases but the interconnectivity of the network decreases with network growth. Overall, the orthographic networks are dense, indicated by a high clustering coefficient as well as a high mean degree.

### Loss of Interconnectivity During Orthographic Learning

In particular, although the number of neighbors per word (mean degree) increases, the overall connectivity of the network decreases as shown by the average path length as well as the clustering coefficient. The interconnectivity of the network slowly decreases between first and second grade, then strongly declines until sixth grade and levels off onwards. That is, in the beginning networks contain a lot of words that are interconnected, thus neighbors of each other. With growing lexicon size, more neighbors are added to already known words but are not necessarily interconnected. This finding appears to be counterintuitive since one would assume a growth of interconnectivity with growing neighborhood sizes. However, regarding orthographic development and learning this makes sense: At the beginning of primary school, children know mostly words that are similar to one another. During development, less similar words are added gradually to the mental lexicon. That is, the mental lexicon becomes more and more differentiated with age.

In addition, the findings can also explain aspects of reading development: At the beginning, in a highly interconnected network, a lot of similar words compete with each other while reading. That is, activation spreads across the network and cannot be focused on the particular target word. It leads to a higher probability of mistakes as well as longer reading times. With growing orthographic lexicon size, the process becomes more refined since the competition between words is minimized due to fewer interconnections in the network. Activation can be centered to the target word, reading thus becomes faster and fewer mistakes are made. This underlines the theories of Metsala (1997) as well as Castles et al. (2007) who suggested a shift from a broader to a more refined process of lexical access. Our findings suggest that the lexicon itself becomes more sophisticated which leads to a more refined access mechanism.

### Implications for the Effect of Neighborhood Size During Development

The change of network measures over time could also imply changes in the effect of neighborhood size and neighborhood frequency over time. In fact, the neighborhoods themselves change with development, the mean degree of our networks increased while the interconnectivity of the neighbors decreased. That is, fitting the findings of Castles et al. (1999), children have smaller neighborhoods than adults which might affect language processing of target words and should be considered in future studies on developmental changes of neighborhood effects. However, as Chan and Vitevitch (2009, 2010) demonstrated, network measures above the traditional neighborhood sizes measure can lead to important findings on influences of lexical structure on language processing. Thus, e.g., the clustering coefficient should also be considered when examining developmental changes in neighborhood effects. While the growth of neighborhoods could lead to the assumption of an increase of neighborhood effects, the decrease of interconnectivity could imply a decline of neighborhood effects. Future research should address this question. In this regard, one important feature of our simulation method is that network measures per grade can be derived, e.g., the mean degree or clustering coefficient for a certain word in a certain age group. Since the estimation of an individual’s lexicon size is possible (see Segbers and Schroeder, 2017), even individual neighborhood sizes could be determined and used for the study of effects in language processing. It has also been shown that the frequency of the neighbors has a crucial moderating effect with high-frequency neighbors having inhibitory and low-frequency neighbors having facilitative effects (Sears et al., 1995; Pollatsek et al., 1999). In our network approach, this frequency information could be implemented using a weighted network approach in which the weights of the paths depend on frequency of the corresponding nodes. This could further help to predict effects of neighbors on lexical access.

### Small World Characteristics in Orthographic Networks

Since we observed a relatively short average path length and a high clustering coefficient, we assume that orthographic networks possess small world characteristics as defined by Watts and Strogatz (1998). As evident from the degree distribution, we also found a scale-free organization of the networks. That is, the orthographic lexicon is structured like semantic and phonological networks with a small number of well-connected nodes which could be denominated as key entries (Borgatti, 2006; Vitevitch and Goldstein, 2014). This fits the findings of several authors who have discussed the existence of a core lexicon which contains words with a lot of connections that are important for communication (e.g., Ferrer et al., 2001; Siew, 2013). Identifying such key players and core lexicons could be promising for teaching strategies since our simulations suggest that they play an important role in language learning.

Throughout all grades, the number of words without orthographic neighbors called “lexical hermits” is constantly high (almost 50%). That is, these words do not connect to the mental lexicon via orthographic similarity. The high proportion reflects the distribution of orthographic neighbors in the German language itself: While because of its morphological richness and orthographic transparency a part of words in German possesses a lot of neighbors, a high proportion of words does not have any orthographic neighbors at all (in childLex, 62.8% of all types do not have neighbors, Schroeder et al., 2015a).

One possible explanation for this in terms of lexical development is that they are learned and integrated via one of the other language domains. Semantic neighbors for example may also play an important role in orthographic learning since words that are semantically connected often appear in the same contexts in written language. Burgess and Lund (2000) have proposed a measure of semantic neighborhood provided by co-occurrence in texts (see also Durda et al., 2006). Analyzing the present data with such a measure of semantic neighborhood could shed more light on the development of the lexical hermits.

Compared to the semantic network described by Steyvers and Tenenbaum (2005) we observed higher values for average and maximum path length as well as for the clustering coefficient and the mean degree in all age groups. This might be due to the fact that the orthographic networks we examined were generally larger than the semantic networks analyzed by Steyvers and Tenenbaum (2005). Furthermore, the results reflect the difference in the definition of neighborhoods: In our study, orthographic neighbors were defined as words with a Levenshtein distance of 1. That is, the probability of a word’s neighbors to also be neighbors is very high in the orthographic domain (e.g., the neighbors *hat* and *fat* for the word *cat* are also neighbors). In the semantic domain, however, neighborhoods are defined by the word’s meaning and are thus more restricted (e.g., although the words *dog* and *mouse* are both semantic neighbors of the word *cat*, they are not necessarily also neighbors of each other). This also results in a higher degree for orthographic then for semantic networks.

In comparison to Vitevitch (2008) phonological network analysis we found higher average path lengths as well as a higher clustering coefficient for all age groups. However, our networks also comprise more nodes than Vitevitch’s analyses. Furthermore, we analyzed the network on a type level which increases the number of neighbors of a word. In addition, differences between languages (English by Vitevitch, German in our study) might also have influenced the results. German is a morphologically rich language (Fleischer et al., 2012) and has a sophisticated inflectional system. This might increase the number of orthographic neighbors. This also holds for the results on the lemma level, since even after lemmatization, German is morphologically rich because of derivation and compounds. A study explicitly comparing different languages – similar to Arbesman et al. (2010) for phonological networks – would be able to address this issue. In general, because of differences in network size and network quality, the comparison of our results to earlier studies on semantic networks (e.g., Steyvers and Tenenbaum, 2005) as well as phonological networks (e.g., Vitevitch, 2008) is not straight forward. For future research, the analysis of networks in different language domains with the same size and quality could lead to more comparable results and findings. Especially the comparison between phonological and orthographic networks could be interesting since both domains share a lot of characteristics in a transparent language such as German.

### Limitations and Future Prospects

Although our results provide an important contribution to the study of orthographic learning, they also have important limitations. Maybe the most important caveat of our study is that the presented findings are not based on real empirical data but on simulations of children’s orthographic development using a corpus sampling approach. Relying on simulations methods is necessary for the analysis of orthographic networks because most of children’s orthographic development takes place after they have entered school and children’s vocabularies are already quite extensive (over 5,000 lemmas or 30,000 types according to our approximations). In contrast to studies focusing on language development in infants (e.g., Hills et al., 2009), it is thus not feasible anymore to collect data for every potential word in the mental lexicon.

The crucial question is whether it is likely that our results will generalize to children’s real orthographic development. Of course, the answer to this question depends on how well the orthographic learning mechanism in the real world is approximated by our sampling procedure and its underlying assumptions. In this context, several points have to be discussed. First, a fundamental assumption of our simulations is that children’s orthographic learning is influenced by probability that a word is encountered in the learning environment, i.e., word frequency. This assumption seems to be plausible given that word frequency is also a major determinant in children’s earlier lexical development (see, e.g., Goodman et al., 2008) and age-of-acquisition norms usually correlate highly with word frequency (e.g., Kuperman et al., 2012). To confirm this, we collected age-of-acquisition data for 1152 words in the childLex corpus (Schröter and Schroeder, 2017) and also found a high correlation between log type frequency and age-of-acquisition norms provided by adults, *r* = -0.51, *p* < 0.001. This confirms that written word frequency is indeed a major factor affecting the time when a word is acquired by children. Naturally, high frequency words are short and possess a lot of neighbors due to the principle of economy in linguistics, that is the aspiration to transport as many information as possible with the smallest effort possible (Vicentini, 2003). Thus, frequency and orthographic neighborhood size highly correlate (*r* = 0.27 in childLex, see also: Landauer and Streeter, 1973; Frauenfelder et al., 1993; Siew, 2013). That is, in our modeled learning process words with a lot of neighbors are also learned first because they also have a higher frequency. The mechanism thus fits other theories on language learning which have shown that words from dense neighborhoods are learned earlier (Storkel, 2004; Hills et al., 2009).

However, there are clearly other factors that influence word learning such as cognitive development, education and personal experience. In addition, other linguistic characteristics such as phonological similarity or semantic concreteness might also influence orthographic learning (Kyte and Johnson, 2006; Ouellette, 2010). These factors should be taken into account in future studies.

Another assumption of the reported simulations is that the childLex corpus that served as the basis for the sampling is a realistic approximation of children’s real print-related learning environment. The childLex corpus is quite extensive compared to other corpora for children and comprises approximately 5 times as many words as an average child is likely to read in grades 1–6. As any corpus, however, it is just a sample from the population of the many books that children potentially can read. During the assembly of the corpus, we took great care to include books that are actually read by the children and based the selection on library loan statistics, teacher ratings, and children’s self-reports (see Schroeder et al., 2015a, for a description of the corpus selection). We are thus confident, that the corpus is an ecologically valid sample for children’s print-related learning environment in German at the beginning of the twenty-first century.

Relatedly, we implicitly assumed that the learning environment stays constant during children’s orthographic learning because we used the same corpus for all grades. This assumption is certainly a simplification, because young children beginning to read are likely to read different books than older children who have different interests and better reading skills. One way to refine our sampling procedure is thus to adapt the learning environment by sampling from different subcorpora for different age groups. However, at least in a transparent orthography such as German, in which most children are able to read rather fluently at the end of grade 1, books for younger and older children are actually not that different in terms of their linguistic characteristics (see Schroeder et al., 2015b, for a summary). Most differences are related to the lexical level, i.e., books for older children have a more varied vocabulary and introduce additional expressions for the same entities. This shift in lexical diversity, however, is taken into account by the frequency-sensitive sampling mechanism. That is, even with the sampling from the subcorpora, we would expect the same pattern of results reported in this study. Finally, we assumed that our virtual participants did not differ in their size of their orthographic networks and all showed the same rate of orthographic growth. Thus, the size of their initial mental lexicon and the number of types that are acquired in each grade were fixed to the average lexicon size and growth rate that has been reported for German (Segbers and Schroeder, 2017). However, it is well known that there are large interindividual differences in children’s print exposure and orthographic development (Stanovich, 2009; Pfost et al., 2014; Schroeder et al., 2015b). It is thus very likely that the size of children’s orthographic networks will show great variability and grow with differential rates. Just simulating orthographic development for average readers is clearly only a starting point for future investigations. An important finding from our study is, however, the mechanisms underlying children’s orthographic network development are remarkably stable and did not differ between children in grades 1 and 8. It is thus rather unlikely that the qualitative nature of children’s orthographic network growth is strongly influenced by mere quantitative aspects of their lexicons. With our findings, we thus provided average numbers on orthographic lexical development for different age groups. However, the next step for future research needs to be the modeling of individual network growth for certain children at different age points over time. Longitudinal data on lexical growth would be necessary to perform these analyses. In addition, in combination with the measurement of the effect of orthographic neighborhood sizes at these different time points these findings could lead to a more sophisticated understanding of orthographic development during primary school.

One further application of our study addresses recommendations for the content of children’s books or texts for children in different age groups. As implicated by our study, young children tend to know a lot of similar words while networks for older children are more differentiated and less connected. Including these known words (and their neighbors) into texts for different age groups could ease the reading process as well as orthographic learning. Clearly, more research on the effect of known neighbors on single word reading is necessary to address this issue.

## Conclusion

To sum up, this study reports data from a simulation study analyzing the development of the orthographic lexicon using graph theory. Our results demonstrate that orthographic networks exhibit small-world characteristics similar to phonological or semantic networks. In addition, we found that the interconnectivity of the network decreases with growth while the neighborhood size itself increases. The results support theories of reading development which claim a shift from a broader to a more fine-grained reading process. Moreover, by showing that the network characteristics and thus neighborhoods change with age, developmental differences in language processing could be explained with our results.

Analyzing orthographic networks using graph theory is thus a promising approach for further research on (individual) language development. In addition, the presented method enables the derivation of age-specific or individual network characteristics which in turn can be used for studies of language processing, namely the effect of network measures on word retrieval.

## Author Contributions

SS and JT contributed to the present paper, discussed the results and implications, and approved the final version to be submitted and agreed to be jointly accountable for all aspects of the work. The study was designed and planned by JT and SS. JT analyzed the data. JT drafted the manuscript and SS critically revised it.

## Conflict of Interest Statement

The authors declare that the research was conducted in the absence of any commercial or financial relationships that could be construed as a potential conflict of interest.
