# Supplementary material for: Orthographic Networks in the Developing Mental Lexicon. Insights From Graph Theory and Implications for the Study of Language Processing
Source: Front Psychol. 2018 Nov 20;9:2252. doi: 10.3389/fpsyg.2018.02252 (PMC6256182; doi:10.3389/fpsyg.2018.02252)
Supplement: Supplementary file 1 [file Table_1.docx]

Supplementary Table 1

*Lexicon sizes and network measures in different age groups for lemmas*

|  | *M* Lexicon Size |  | Network Measures *M (SD)* ** | | | | | |
| --- | --- | --- | --- | --- | --- | --- | --- | --- |
| Grade | Lemmas |  | *n* | *<k>* | *L* | *D* | *C* |  |
| 1 | 5925 |  | 1922 (25) | 4.79 (0.14) | 10.81 (1.09) | 32.00 (4.18) | .61 (.04) |  |
| 2 | 6097 |  | 1980 (26) | 4.81 (0.14) | 10.79 (0.98) | 31.84 (3.41) | .61 (.05) |  |
| 3 | 11182 |  | 3758 (41) | 5.50 (0.13) | 9.94 (0.35) | 30.02 (2.71) | .64 (.04) |  |
| 4 | 14819 |  | 5021 (48) | 5.87 (0.11) | 9.57 (0.22) | 30.28 (2.62) | .65 (.03) |  |
| 5 | 18812 |  | 6378 (52) | 6.21 (0.13) | 9.39 (0.20) | 31.68 (3.11) | .66 (.03) |  |
| 6 | 25694 |  | 8651 (62) | 6.67 (0.10) | 9.16 (0.20) | 33.58 (4.74) | .66 (.02) |  |
| 8 | 38029 |  | 12589 (61) | 7.12 (0.06) | 9.09 (0.27) | 38.02 (6.02) | .63 (.01) |  |
